# Diagnostic Reference Level of Radiation Dose and Image Quality among Paediatric CT Examinations in A Tertiary Hospital in Malaysia

**DOI:** 10.3390/diagnostics10080591

**Published:** 2020-08-14

**Authors:** Nor Azura Muhammad, Muhammad Khalis Abdul Karim, Hasyma Abu Hassan, Mazliana Ahmad Kamarudin, Jeannie Hsiu Ding Wong, Kwan Hoong Ng

**Affiliations:** 1 Department of Physics, Faculty of Science, Universiti Putra Malaysia, Serdang 43400, Selangor, Malaysia; norazura11@gmail.com (N.A.M.); mazliana_ak@upm.edu.my (M.A.K.); 2 Centre of Diagnostic Nuclear Imaging, Faculty of Medicine, Universiti Putra Malaysia, Serdang 43400, Selangor, Malaysia; 3 Department of Imaging, Faculty of Medicine and Health Sciences, Universiti Putra Malaysia, Serdang 43400, Selangor, Malaysia; hasyma@upm.edu.my; 4 Department of Biomedical Imaging, University Malaya Medical Centre, Petaling Jaya 59100, Kuala Lumpur, Malaysia; jeannie.wong@ummc.edu.my (J.H.D.W.); ngkh@ummc.edu.my (K.H.N.); 5 Department of Medical Imaging and Radiological Sciences, College of Health Sciences, Kaohsiung Medical University, Kaohsiung 80708, Taiwan

**Keywords:** paediatric CT, CT dose optimization, diagnostic reference level, noise index, image quality

## Abstract

Pediatrics are more vulnerable to radiation and are prone to dose compared to adults, requiring more attention to computed tomography (CT) optimization. Hence, diagnostic reference levels (DRLs) have been implemented as part of optimization process in order to monitor CT dose and diagnostic quality. The noise index has recently been endorsed to be included as a part of CT optimization in the DRLs report. In this study, we have therefore set local DRLs for pediatric CT examination with a noise index as an indicator of image quality. One thousand one hundred and ninety-two (1192) paediatric patients undergoing CT brain, CT thorax and CT chest-abdomen-pelvis (CAP) examinations were analyzed retrospectively and categorized into four age groups; group 1 (0–1 year), group 2 (1–5 years), group 3 (5–10 years) and group 4 (10–15 years). For each group, data such as the volume-weighted CT dose index (CTDI_vol_), dose-length product (DLP) and the effective dose (E) were calculated and DRLs for each age group set at 50th percentile were determined. Both CT dose and image noise values between age groups have differed significantly with *p*-value < 0.05. The highest CTDI_vol_ and DLP values in all age groups with the lowest noise index value reported in the 10–15 age group were found in CT brain examination. In conclusion, there was a significant variation in doses and noise intensity among children of different ages, and the need to change specific parameters to fit the clinical requirement.

## 1. Introduction

The introduction of the Computed Tomography (CT) scanner in 1972 allowed a combination of a series of X-ray images to generate high contrast sectional images of the human anatomy. Since then, it has contributed to the accurate diagnosis and case management of many patients, allow surgeons to conduct complicated operations. However, the utilization of CT has increased in frequency, to the extent that safety concerns have been raised for patients, especially children, as they may be exposed to dangerous levels of ionizing radiation during the examinations [1]. This trend will continuously be observed and likely to develop in the coming future. CT scan previously has been linked to causing severe deterministic effect as well as the stochastic effect due to unoptimized practiced delivered [2]. According to the report by Ogbole (2011), in the last three to five years, the use of CT had increased globally [3]. For example, in Japan, a 1996 survey on the number of CT scanners in use for the country’s population was higher compared with the USA. Furthermore, a report by Shrimpton et al. (2006) quoted a 1989 British survey that found approximately four per cent of CT examinations in the United Kingdom were performed on children below 15 years old [4].

The estimated dose for children in CT scans has become an area of concern with increasing awareness of radiation risk [4]. The United Nations Scientific Committee on the Effects of Atomic Radiations (UNSCEAR) reported in 2010 that the tissues and organs of children were more radiosensitive compared with adults, and they are more at risk of developing radiation-induced cancer after a long latency period [5]. Hence, the risk of CT radiation exposure in children should be justified and optimized according to the “As Low As Reasonably Achievable” (ALARA) principle. However, several studies have found large differences in radiographic imaging procedures at different hospitals, prominent to varying degrees of radiation exposure on patients [6,7].

In 1990 the International Commission for Radiological Protection (ICRP) launched “diagnostic reference level” (DRL) in order to urge authorities, governing bodies and health institutions in medical practice to establish safety standards for radiation exposure that conform to clinical purposes [5]. DRLs are designed to represent the safety reference of radiological procedures for a local institute, established imaging centre, specific region or even nation. The derivation of DRLs allows the institution to control the use of radiological procedures in a way that suits health needs and eliminates undesired exposure without compromising image quality. Generally, the DRL has been proposed as the 75th percentile (third quartile) of the national dose apportioning ever since it was first established by the ICRP in 1996. Noting that, professionals or regulatory bodies may determine a national DRL from wide-ranging surveys at hospitals in a region or country [6,7].

The European Guidelines on DRLs for pediatric imaging (PiDRL) states that DRLs may be used to optimize CT radiation dose without compromising image quality or care in paediatric patients [8]. The age and weight must be taken into account when establishing DRL for paediatric patients, owing to large variations in the children’s body size. The association between dose and patient size may be used to adjust CT protocols for a specific patient [9]. Standardized CT measurements used to set up DRLs are the volume CT dose index (CTDI_vol_) and dose-length product (DLP) [10,11]. Several studies reported that DRL had been used in certain countries for quality assurance and CT dose optimization [12,13]. As well as exposure to radiation, image quality is also important and must be taken into account in all optimization processes [14]. The action taken can be considered as the very first phase towards extending the DRL definition.

Dose and image quality are two leading indicators that reflects the reference points that help radiology personnel to seek and optimize CT technique. This research thus establishes a local DRL through radiation exposure and image quality assessment in popular pediatric CT studies. Specifically, DLPs and image noise will be added to improve DRLs further.

## 2. Materials and Methods

This retrospective study was approved by our Medical Research Ethics Committee (MREC) of University of Malaya Medical Center (approval no: MREC ID NO 2018920-6690, date: 7-Jan-2019) and the requirement for informed consent has been waived.

Data on pediatric cases from January 2012 to December 2018 were obtained from the Biomedical Imaging Department, UMMC through the Picture Archiving Communication System (PACS). Only CT brain, CT thorax and CT chest-abdomen-pelvis (CAP) performed on pediatric patients was included in this study. Subjects with multiple examination and insufficient information were excluded from the data subjects. All subjects were categorized into four age groups: Group 1 (0–1 year), Group 2 (1–5 years), Group 3 (5–10 years) and Group 4 (10–15 years).

### 2.1. CT Acquisition Data Collection

This study included two types of CT scanners, a 64-slice CT Somatom Concept (Siemens Healthineers, Erlangen, Germany) with moderate soft reconstruction kernel (B30f) Filter Back Projection (FBP), and 128 multi-slice CT Ingenuity Core (Philips Koninsklijke, Amsterdam, The Netherlands) with iterative reconstruction technique software (Ingenuity-128 with iDose^4^). Pertinent scanning data comprising effective tube current (mAs), tube voltage (kVp), exposure time (s), table feed (TF), pitch, scan length and slice thickness were retrieved from the hospital’s Digital Imaging and Communications in Medicine (DICOM) system. CTDI_vol_ and DLP of CT brain, CT thorax and CT CAP examinations, with and without contrast enhancement, were extracted from the PACS console. Most examinations were performed with automatic tube current modulation (TCM), which lessen radiation exposure in terms of Z-axis.

### 2.2. Radiation Dose Calculation Software

Input such as scanner model, manufacturer and scanning parameters were provided, prior the estimation by CT before CTDI_vol_, DLP and E measurement. Furthermore, CTDI_vol_ and DLP were premeditated and validated by the CT-EXPO software Version 2.3.1 (Sascrad, Berlin, Germany). Also, the software was used to calculate the effective dose (E) based on the tissue weighting factor published in ICRP 60 in 1991 and ICRP 103 in 2007.

### 2.3. Image Noise Evaluation

Noise value was objectively evaluated using the Radiant DICOM Viewer software (Medixant, Poznan, Poland) to represent the image quality indicator. Quality assessment was performed by putting the same size of regions of interest (ROI), approximately 0.8 cm^2^ on the gray matter area of frontal lobe for CT brain, on the pulmonary trunk of CT thorax and the liver area of CT CAP images. All the CT number and standard deviation (SD) were recorded in Hounsfield Units (HU). Limited photons would cause the noise in its imaging in the X-ray tube. The noise presenting the metric number of the pixel value and the output of the diagnostic image noise was calculated as follows:

(1) $$SD\;\left(\sigma \right)=\sqrt{\frac{\sum^{\;}{\left(x_{ip}-x_{m}\right)}^{2}}{t-1}}$$

where *x_ip_* represents the single pixel value, *x_m_* is the average of all the pixel values in the ROI and *t* is the total of t pixel amounts in the ROI.

### 2.4. Data Analysis

Data were presented descriptively using the mean, median, range (min-max) and interquartile (IQ) values for each pediatric age group based on region and protocol used. The 75th percentile was used to compare with the established DRL from previous studies. Statistical analysis was performed using SPSS Version 25.0 (IBM Corporation, Armonk, NY, USA), and significant differences in radiation doses and noise level between age groups were determined when *p* < 0.05. The Kolmogorov–Smirnov test was used to determine the normality of data distribution and Kruskal–Wallis H test was used to determine differences between age groups.

## 3. Results

**Figure 1:**
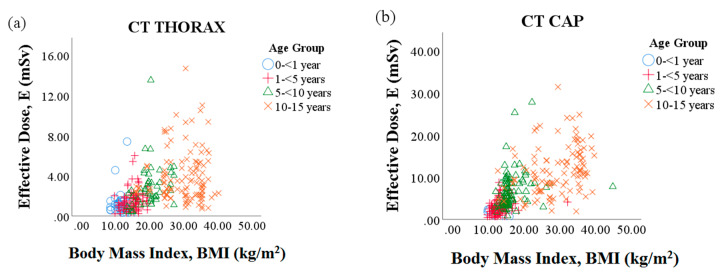
The positive correlation between effective dose (E) and body mass index in paediatric CT examination in each age category. (**a**) CT thorax (r = 0.47) (**b**) CT CAP (r = 0.65).

Of the 1192 cases in this study, 50% (599 patients) were CT brain, 24% (287 patients) were CT thorax, and 26% (306 patients) were CT CAP patients. Although the Philips Ingenuity Core 128 multi-slice scanner was installed in the institution only in 2015, a larger number of CT brain cases (569 cases, 95%) have been used. Noting that all cases from the scanner have been applied with level 4 of iterative reconstruction algorithm, iDose^4^. Meanwhile, the Siemens Somatom Definition AS+ dual-source 64 MDCT scanner, which was installed in 2009, was used to scan 96% (275 cases) of CT thorax and 96.5% (295 cases) of CT CAP cases. Table 1 shows the patient characteristics in terms of body weight, height and body mass index (BMI) according to scan and age groups. It could be observed that males were a majority, and Groups 2 and 4 had the most patients. Figure 1 illustrates the relationship between BMI and E in CT thorax and CT CAP. Both E exhibit a linear relationship with the subject’s BMI. Table 2 tabulate scanning acquisition parameter that are related to image noise and radiation dose received by pediatric patients in all CT protocols. The effective mAs and tube potential (kVp) of the pediatric patients varied among age groups for all three CT examinations. The pitch setting in the CT brain was lower than one (<1) compared to CT CAP and CT thorax in all the age groups. However, the effective mAs in CT brain was notably higher than other examinations.

As summarized in Table 3, the 50th percentile (median) and mean values of CTDI_vol_ and DLP for CT brain were notably higher compared to CT thorax and CT CAP. The E value for CT brain and CT thorax in Group 1 had increased compared to Groups 2 and 3. The effective dose value in CT CAP were higher in the oldest age group (Group 4) compared to younger ones. Nevertheless, the cumulative effects of the dosage level revealed that older age groups (Group 4) consistently obtained a higher radiation dose in all CT examinations. The statistical test found significant differences in CT radiation dose metrics in terms of CTDI_vol_, DLP and E between all age groups in all CT examinations. For image noise, the median and mean values were substantially different between CT examinations. However, no significant differences in the standard deviations were observed in CT CAP and CT brain.

**Figure 2:**
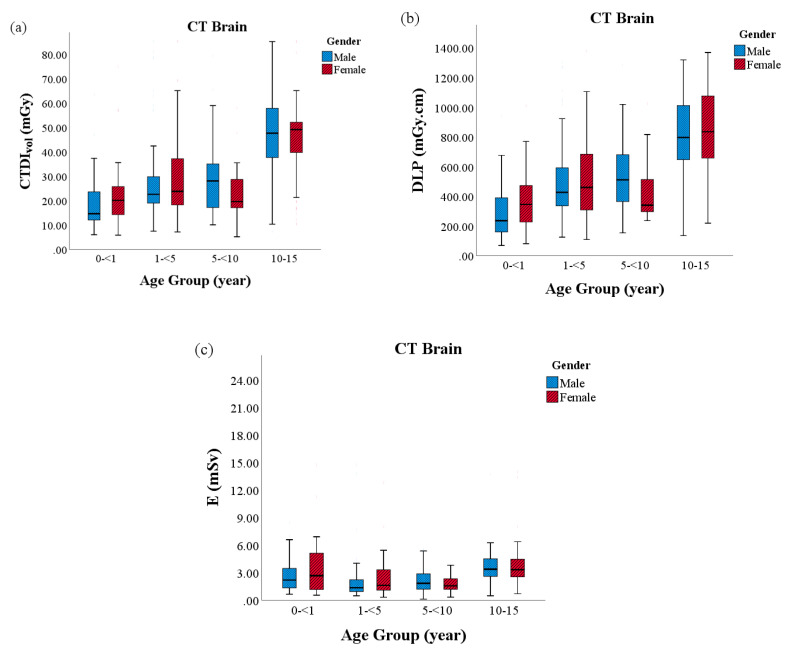
The radiation doses between sex and age in CT brain: (**a**) volume computed tomography dose index (CTDI_vol_); (**b**) dose length product (DLP); and, (**c**) effective dose (E).

**Figure 3:**
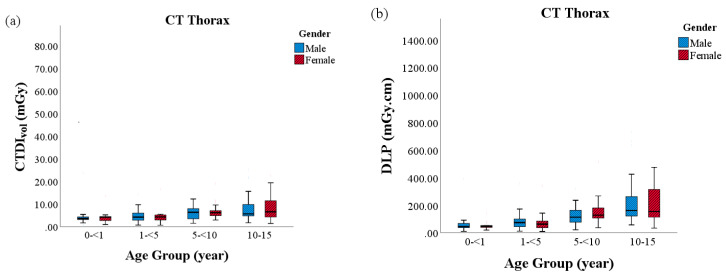

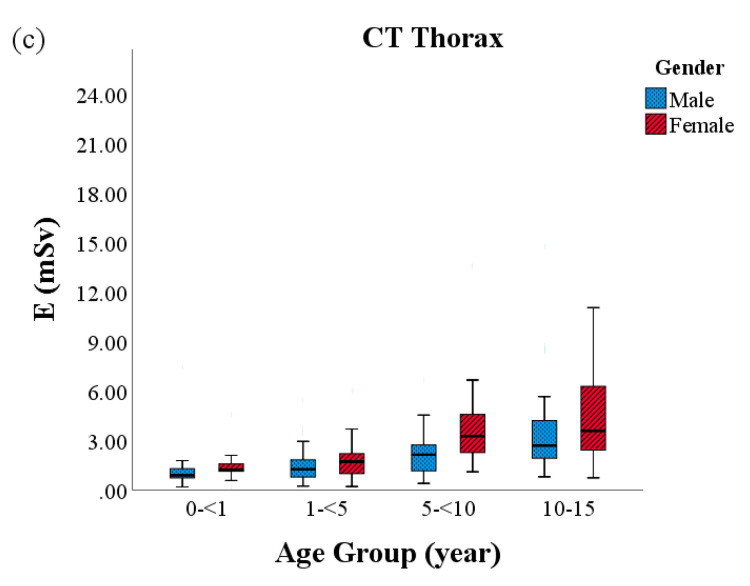
The radiation doses between sex and age in CT thorax: (**a**) volume computed tomography dose index (CTDIvol); (**b**) dose length product (DLP); and, (**c**) effective dose (E).

**Figure 4:**
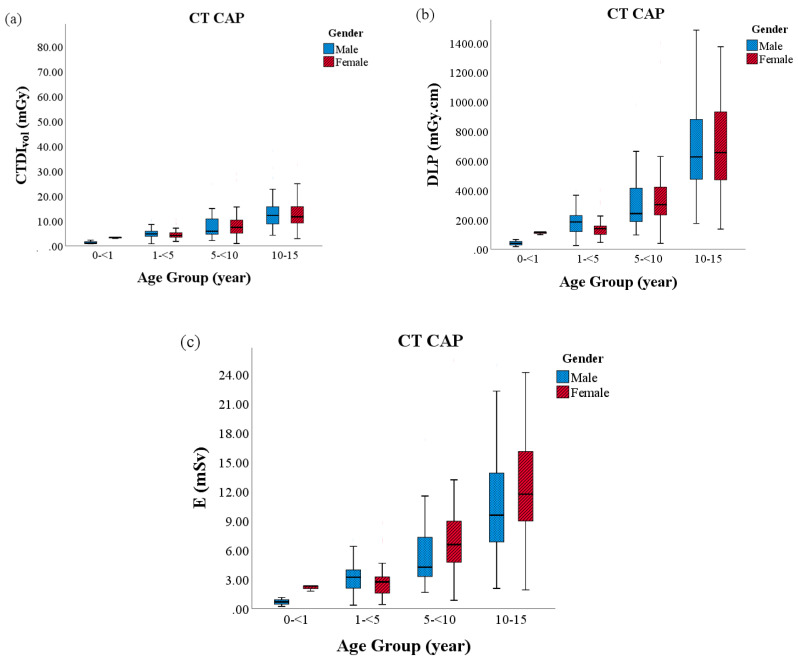
The radiation doses between sex and age in CT CAP: (**a**) volume computed tomography dose index (CTDIvol); (**b**) dose length product (DLP); and, (**c**) effective dose (E).

The variation in the CTDI_vol_, DLP and E as stated by the age and gender of the patient in CT brain, CT thorax and CT CAP are shown in Figure 2, Figure 3 and Figure 4, respectively. CT brain continued to show substantially different inter-quartile range for all radiation dose metric evaluations between the youngest and oldest age groups. Nevertheless, effective dose values were observed highest in CT CAP and CT thorax examinations, particularly in Group 4. The median exposure between the genders, albeit females in Group 1 and Group 4, appeared to be receive more exposure in all CT protocols.

Table 4 summarizes the reference levels that represent the median value of CTDI_vol_, DLP and E, as well as the median values of image noise. The reference ranges referred to the difference between the 25th percentile (first quartile) and 75th percentile (third quartile) of the dose and image noise. As per recommendation in ICRP 103 and National Council on Radiation Protection Measurement (NCRP) Report 172, the local DRL was defined as the 50th percentile (median) value of each CT protocol for pediatric patient groups in this study. Table 5 provides a comparison with other local and international pediatric DRL studies, between the 75th percentile of the CTDI_vol_ and DLP analysis. The findings of CTDI_vol_ were similar to German, United States, Korea and European studies for CT Brain and CT thorax [8,15,16,17,18]. However, DLP values for CT brain were significantly higher than others study except for reference level from Jordan [19].

## 4. Discussion

Both developed and developing countries have established dose survey data as a guideline to develop their own DRL in medical imaging procedures [20,21,22]. A DRL could serve as a good tool in optimizing the radiation doses of CT examinations in pediatric patients and ensuring good image quality [23,24,25]. The Malaysian Health Ministry had established its DRL for radiological procedures in its Medical Radiation Exposure Report in May 2013. However, the DRL for pediatric patients was still incomplete due to the lack of data. Hence, this study aimed to close the gap by evaluating the radiation dose and image quality (noise) of CT brain, CT thorax and CT CAP from a single healthcare institution to establish localized DRLs for pediatric patients. Children’s weight and size is highly variable compared with adults. According to the PiDRL and ICRP documents 135, the DRLs for children are proposed to be defined according to weight (5 kg, 10 kg, 20 kg, 30 kg, 40 kg, 50 kg and 60 kg), classes of weight (<10 kg, 10–15 kg, 15–30 kg, 30–60 kg, 60 kg) or age groups.

The radiation dose exposure also varied by parameters and protocols, including tube voltage (kVp), effective mAs, pitch and slice thickness [26,27,28]. That is why the CTDI_vol_ and the DLP in the CT brain were higher than the CT CAP and CT thorax in all ages. In the meantime, E was higher in CT brain and CT thorax groupings in this analysis. This was in line with the study by Aw–Zoretic et al. (2014), in the younger age groups, where the E in CT brain in contrast with the older age groups was higher. This contributed to safety concerns as E was a factor in the assessment of the biological effects of radiation on patients [29]. Recent studies have confirmed a strong correlation between CT and carcinogenic risk radio-exposure, particularly in pediatric patients [9,30,31].

Despite a lesser radiation exposure in the youngest age group (Group 1) due to CT parameter setting, a higher noise value was also observed in the same group. The proper setting of CT scanning parameters, such as tube voltage, current, pitch and slice thickness could affect image quality [32]. A study by Paolicchi et al. (2014) stated that the effective training of radiographers in adjusting CT parameters could reduce radiation dose and image noise. The higher DLP in CT brain could be mainly due to the over-ranging beam when scanning was performed, as the examinations mainly involved accident and trauma cases.

In spite of that, multiple studies had stated that it was challenging to balance radiation exposure and image quality due to variances in the patients’ body habitus [33,34]. Therefore, adjustments of CT scanning parameters should be made in the optimization processes, particularly when pediatric patients were involved. The noise reference level and range were included when determining the DRL values of this study. Therefore, it could be considered an expanded concept of DRL as suggested by IAEA in 2018, and acts as a guide in balancing the radiation exposure and image quality to identify the recognized range of radiation dose with adequate image quality.

Additionally, the reference level and range of radiation dose and image noise could be helpful in the investigation of unnecessary radiation dose and poorer image quality as the guidance information for the optimization procedure. ICRP Publication 135 also stated that similar to DRL standards of radiation dose, image quality was also the crucial part of an optimization study.

The 75th percentile (third quartile) CTDI_vol_ and DLP values in this study were compared with the DRL data from studies in other countries. The DLP results in CT brain were slightly higher than the studies in USA and Switzerland [15,17]. The higher DLP in CT brain could mainly be due to the over-ranging beam when scanning was performed. The data of the radiation dose in CT thorax and CT CAP was notably lower than the Jordanian study by Rawashdeh et al. (2019). This difference could be due to the selection mode of acquisition axial or helical scanning, CT scanning parameter settings and different manufacturers of the CT scanners used [14].

The image noise in CT examinations was one of the indispensable factors in image quality valuation. To date, references and guidelines for the image quality still lacking in CT examinations, especially on noise magnitude. The level of tolerable noise, therefore, depended on the ability of an experienced radiologist [35]. Nowadays, most CT scanners come equipped with iterative reconstruction algorithms to reduce image noise. A study by Pien et al. (2011) stated that applying iterative reconstruction in an objective and subjective image quality assessments resulted in reduced image noise even with low tube voltage being used [36,37]. The mean image noise in this study’s CT brain was in line with Park et al. (2017), and was within a plausible range. With the advancement of artificial intelligence, iterative reconstruction with deep learning-based image has significantly reduce radiation dose and improve image quality. A recent study has demonstrated that deep learning algorithm can achieve better image quality than commercially available iterative reconstruction among three leading vendors [38]. Therefore, the framework may be widely available in the future if it is proof that it is appropriate for the optimization process.

This study had several limitations. First, the estimated results were collected from just one institution in the country and only involved pediatric data. Future studies could consider including adult patients as an extra group and increase the number of healthcare institutions involved. Second, CT protocols used were from the same scanner model to avoid variations in acquisition parameters. Third, this study only evaluated image noise parameters in image quality assessments. Future studies should consider other parameters of objective image quality, and also include subjective image quality evaluation. Lastly, this study only categorized the pediatric subjects into age groups. It is recommended to group the patients in other ways, such as weight as recommended by ICRP.

## 5. Conclusions

To conclude, radiation exposure and image noise vary widely among children of different ages, and there might be a need to establish DRLs for specific age groups. DRLs were recognized as an important tool in optimizing radiation dose for pediatric patients according to the ALARA principle. Furthermore, children were more sensitive to radiation compared to adults because of their developing organs and tissues, and the longer post-exposure life expectancy would increase their lifetime risk of developing radiation-induced malignancies. Thus, the established data on the dose and noise reference level in this study could contribute as a guide to optimize pediatric CT.

## Figures and Tables

**Table 1:** Patient characteristic distribution of CT brain, thorax and CAP by sex and age groups.

CT Protocol	Parameters					
	Age Group (Years)	Sex	N	Weight (kg) *	Height (cm) *	BMI (kg m^−2^) *
CT Brain	Group 1 (0–<1)	M	62	4.70 ± 1.4	42.95 ± 8.9	10.99 ± 1.5
		F	32	4.76 ± 1.4	41.91 ± 11.4	13.23 ± 1.4
	Group 2 (1–<5)	M	78	11.27 ± 3.4	75.93 ± 13.6	14.62 ± 3.2
		F	67	12.62 ± 2.5	94.63 ± 14.2	13.23 ± 1.4
	Group 3 (5–<10)	M	57	24.82 ± 6.1	124.38 ± 10.5	19.42 ± 4.6
		F	39	17.67 ± 1.3	116.40 ± 7.5	15.20 ± 1.0
	Group 4 (10–15)	M	164	49.14 ± 8.9	147.54 ± 8.6	33.24 ± 5.5
		F	100	54.31 ± 6.0	158.40 ± 11.7	34.26 ± 2.7
CT Thorax	Group 1 (0–<1)	M	25	5.09 ± 1.1	43.76 ± 9.8	11.72 ± 1.6
		F	13	4.62 ± 1.4	41.62 ± 13.0	11.26 ± 1.6
	Group 2 (1–<5)	M	63	12.39 ± 2.6	93.04 ± 8.3	14.75 ± 2.9
		F	22	13.38 ± 2.2	86.09 ± 2.1	15.53 ± 2.0
	Group 3 (5–<10)	M	29	23.00 ± 5.7	120.77 ± 10.6	21.47 ± 2.8
		F	22	27.65 ± 4.2	128.57 ± 6.7	18.98 ± 4.1
	Group 4 (10–15)	M	56	48.82 ± 8.6	161.65 ± 11.0	30.10 ± 4.4
		F	57	41.62 ± 11.5	155.37 ± 11.2	29.96 ± 6.7
CT CAP	Group 1 (0–<1)	M	3	4.35 ± 0.7	43.20 ± 3.4	13.39 ± 2.3
		F	4	5.73 ± 0.5	41.18 ± 3.4	10.54 ± 1.0
	Group 2 (1–<5)	M	55	12.52 ± 6.1	87.54 ± 15.2	13.91 ± 3.2
		F	40	11.06 ± 2.1	88.27 ± 12.8	12.50 ± 1.1
	Group 3 (5–<10)	M	39	20.49 ± 8.1	110.91 ± 7.0	17.63 ± 13.2
		F	55	19.93 ± 17.1	116.57 ±10.8	17.27 ± 5.0
	Group 4 (10–15)	M	59	47.31 ± 12.4	151.27 ± 16.4	28.03 ± 6.7
		F	51	42.98 ± 13.2	154.75 ± 11.9	30.24 ± 6.7

* Mean ± D, M = Male, F = Female, N = Number.

**Table 2:** Parameters used in CT brain, thorax, and CAP.

Parameters	CT Protocol											
	CT Brain				CT Thorax				CT CAP			
Age Group (years)	Group 1	Group 2	Group 3	Group 4	Group 1	Group 2	Group 3	Group 4	Group 1	Group 2	Group 3	Group 4
	(0–<1)	(1–<5)	(5–<10)	(10–15)	(0–<1)	(1–<5)	(5–<10)	(10–15)	(0–<1)	(1–<5)	(5–<10)	(10–15)
Tube Voltage (kVp)	100	100	100,120	120	80,100	100	100,120	120	80	100	100,120	120
Effective Tube current (mAs) *	209.10 ± 27.0 (80–380)	248.23 ± 35.9 (90–423)	185.50 ± 20.5 (20–400)	233.33 ± 24.8 (80–430)	74.37 ± 44.8 (27–225)	76.80 ± 34.2 (10–261)	110.81 ± 54.5 (35–225)	145.28 ± 52 (20–360	37.10 ± 5.2 (26–40)	76.80 ± 34.2 (28–177)	110.81 ± 54.5 (35–272)	145.28 ± 52.0 (53–287)
Scan Range *	11.91 ± 4.2	14.42 ± 1.6	14.53 ± 4.8	15.03 ± 4.7	12.22 ± 2.7	16.98 ± 15.2	18.81 ± 5.4	25.01 ± 5.2	28.92 ± 4.6	31.49 ± 1.6	40.77 ± 4.6	52.25 ± 10.5
Pitch	0.4–0.64	0.4–0.64	0.4–0.64	0.4–0.64	1–1.4	1–1.4	1–1.4	1–1.4	1.4	1.4	1.2–1.4	1.2–1.4
Slice thickness (mm)	3	3	3	3	0.8–5	3–5	3–5	3–5	3	3	3–5	3
Collimation	40 × 0.6	40 × 0.6	40 × 0.6	40 × 0.6	64 × 0.6	64 × 0.6	64 × 0.6	64 × 0.6	64 × 0.6	64 × 0.6	64 × 0.6	64 × 0.6
Table feed	11–16	11–16	11–16	11–16	26.9	26.9	26.9	26.9	26.9	26.9	26.9	26.9

* Mean ± SD (Min–Max).

**Table 3:** Summary of radiation dose and noise value.

CT Procedure	Age Group (years)	CTDIvol (mGy)			DLP (mGy.cm)			E (mSv)			Noise Value (HU)		
		Mean	Median	Range (Min-Max)	Mean	Median	Range (Min–Max)	Mean	Median	Range (Min–Max)	Mean	Median	Range (Min–Max)
Brain	Group 1 (0–<1)	27.2	22.64	3.6–123.1	308.9	250.1	69–1010	3.0	2.4	0.6–14.7	4.0	3.6	2.8–6.4
	Group 2 (1–<5)	38.6	29.39	4.4–123.4	510.7	449.0	109–1379	2.2	1.5	0.3–14.7	3.2	3.1	1.5–6.2
	Group 3 (5–<10)	40.6	31.63	2.8–122.2	493.0	458.5	154–1285	2.1	1.7	0.1–5.5	3.8	3.3	2.6–5.5
	Group 4 (10–15)	69.1	68.61	10.0–124.4	811.4	814.1	135–1779	3.6	3.3	0.5–14.0	3.5	3.6	1.9–5.1
	*p*-value	<0.001			<0.001			<0.001			0.184		
Thorax	Group 1 (0–<1)	4.3	3.7	0.8–23.9	59.0	46.9	10.5–393.6	1.3	1.0	0.2–7.4	21.9	23.1	8.7–29.0
	Group 2 (1–<5)	4.6	4.2	0.6–16.7	79.5	66.7	9.3–358.1	1.6	1.4	0.2–6.0	21.0	22.5	12.8–29.1
	Group 3 (5–<10)	6.6	6.2	1.5–19.0	135.3	126.3	21.6–516.5	2.9	2.5	0.4–13.5	21.6	21.8	10.2–22.5
	Group 4 (10–15)	8.0	6.2	1.4–25.2	207.7	155.5	34.0–732.0	4.0	3.1	0.7–14.7	13.3	12.7	6.8–26.9
	*p*-value	<0.001			<0.001			<0.001			0.042		
CAP	Group 1 (0–<1)	2.5	3.0	0.8–3.4	81.1	98.1	17.2–115.6	1.6	1.8	0.2–2.3	11.2	11.3	6.8–16.3
	Group 2 (1–<5)	4.9	4.6	0.8–10.4	164.1	150.2	24.6–400.1	3.0	2.9	0.4–8.8	10.5	10.2	4.1–21.8
	Group 3 (5–<10)	8.2	7.1	1.0–28.8	347.0	292.6	39.7–1398.6	6.8	5.8	0.9–27.8	11.9	9.3	6.2–20.9
	Group 4 (10–15)	13.1	11.7	2.9–38.0	705.3	644.9	136.5–2223.2	11.7	11.1	1.9–31.5	9.9	9.8	7.0–13.4
	*p*-value	<0.001			<0.001			<0.001			0.898		

**Table 4:** Dose reference levels and ranges of CTDI_vol_, DLP, and E and noise reference levels and ranges in CT brain, CT thorax and CT CAP.

CT Procedure	Age Group (years)	CTDIvol(mGy)		DLP (mGy.cm)		E (mSv)		Noise Value (HU)	
		Dose Reference Level	Dose Reference Range	Dose Reference Level	Dose Reference Range	Dose Reference Level	Dose Reference Range	Noise Reference Level	Noise Reference Range
Brain	Group 1 (0–<1)	22.64	12.5–24.1	250.1	172.3–426.5	2.4	1.3–3.8	3.6	3.3–4.5
	Group 2 (1–<5)	29.39	18.4–34.0	449.0	316.0–622.0	1.5	1.0–2.4	3.1	2.5–3.2
	Group 3 (5–<10)	31.63	17.2–32.1	458.5	324.0–645.5	1.7	1.2–2.6	3.3	3.0–4.7
	Group 4 (10–15)	68.61	39.7–57.8	814.1	657.5–1040.5	3.3	2.6–4.5	3.6	2.7–4.3
Thorax	Group 1 (0–<1)	3.7	2.9–4.4	46.9	37.7–67.8	1.0	0.7–1.5	23.1	19.3–26.1
	Group 2 (1–<5)	4.2	2.9–5.4	66.7	42.9–97.1	1.4	0.8–2.0	22.5	14.6–26.0
	Group 3 (5–<10)	6.2	4.3–7.8	126.3	85.6–163.2	2.5	1.7–3.4	21.8	14.2–26.1
	Group 4 (10–15)	6.2	4.7–10.5	155.5	119.3–283.5	3.1	2.2–5.3	12.7	8.84–15.8
CAP	Group 1 (0–<1)	3.0	1.7–3.4	98.1	52.7–115.6	1.8	1.0–2.4	11.3	8.3–14.0
	Group 2 (1–<5)	4.6	3.7–5.7	150.2	115.6–204.0	2.9	2.0–3.8	10.2	8.5–11.2
	Group 3 (5–<10)	7.1	4.9–10.6	292.6	208.7–419.4	5.8	4.0–8.6	9.3	7.8–15.5
	Group 4 (10–15)	11.7	8.9–15.8	644.9	472.0–911.1	11.1	7.6–15.1	9.8	9.4–10.3

**Table 5:** Comparison of the third quartile (75th percentile) value with established diagnostic reference level (DRL) from other studies.

CT Procedure	Age Group (Years)	This Study		Gao et al. (2018)		Rawashdeh et al. (2019)		Verdun et al. (2008)		Galanski et al. (2005)		EC (2015)	
		CTDI_vol_	DLP	CTDI_vol_	DLP	CTDI_vol_	DLP	CTDI_vol_	DLP	CTDI_vol_	DLP	CTDI_vol_	DLP
Brain	Group 1 (0–<1)	24	427	26	340	49	744	20	270	34	393	25	370
	Group 2 (1–<5)	34	622	35	558	55	982	30	420	49	611	38	505
	Group 3 (5–<10)	32	646	38	607	65	1130	40	560	58	711	53	700
	Group 4 (10–15)	58	1041	48	815	61	1207	60	1000	65	920	60	900
Thorax	Group 1 (0–<1)	4	68	1	28	5.6	124	5	110	7	93	3	80
	Group 2 (1–<5)	5	97	4	101	7.4	222	8	200	8	137	6	115
	Group 3 (5–<10)	8	163	5	153	12.9	416	10	220	12	257	6	180
	Group 4 (10–15)	11	283	7	230	12.9	496	12	460	16	488	7	200
CAP	Group 1 (0–<1)	3	116	-	-	16.1	509	-	-	-	-	-	-
	Group 2 (1–<5)	6	204	-	-	16.1	787	-	-	-	-	-	-
	Group 3 (5–<10)	11	419	-	-	12.9	702	-	-	-	-	-	-
	Group 4 (10–15)	16	911	9	637	16.1	755	-	-	-	-	-	-
